# Does Patient Sex Influence the Symptom Pattern for Patients With Gastro‐Oesophageal Reflux Disease and the Response of Symptoms to Fundoplication?

**DOI:** 10.1111/ans.70152

**Published:** 2025-04-23

**Authors:** Joshua N. Hammerschlag, Ross H. Roberts, Andrew McCombie

**Affiliations:** ^1^ Christchurch Hospital, Te Whatu Ora Health Christchurch New Zealand

**Keywords:** female, fundoplication, gastro‐oesophageal reflux, heartburn, upper gastrointestinal tract

## Abstract

**Background:**

Several studies show that female patients experience lower satisfaction after anti‐reflux surgery. We hypothesised that there is a pattern of symptoms common to female patients presenting with GORD and that it is these symptoms in particular that lead to inferior outcomes. We also hypothesised that these symptoms would respond differently to the different types of fundoplication.

**Methods:**

Pre‐operative and post‐operative questionnaires for all patients undergoing laparoscopic fundoplication were analysed. Pre‐operative symptoms, improvement of symptoms, patient demographics and type of fundoplication were assessed.

**Results:**

The study evaluated 328 patients, 156 females and 172 males. Eighty‐seven females (55.8%) and 135 males (78.5%) underwent a Nissen Fundoplication (*p* < 0.001) with the rest undergoing a posterior 270° (partial) fundoplication. Females reported most symptoms more frequently than males, and a predominantly female pattern of symptoms was identified. Bloating (*p* ≤ 0.001), asthma (*p* = 0.045), constipation (*p* = 0.010) and diarrhoea (*p* = 0.023) were significantly more associated with being female. At 18 months post‐surgery, outcomes for patients presenting with ‘female‐pattern’ symptoms were not improved by undertaking partial fundoplication when compared to Nissen fundoplication. The only symptom that was affected by the type of fundoplication was dysphagia, with a significant number of patients experiencing worsening of dysphagia after a Nissen fundoplication (*p* < 0.01).

**Conclusion:**

There are certain symptoms that affect female patients who present with GORD more commonly than male patients, and this may contribute to lower satisfaction outcomes. Partial fundoplication does not appear to offer a significant advantage in improving these symptoms.

## Graphical abstract

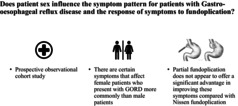

## 1 Introduction

From its inception in 1991, laparoscopic fundoplication has delivered excellent results in the management of gastro‐oesophageal reflux disease (GORD) [1]. It is a safe and effective surgery, which provides long‐term treatment of the symptoms of GORD. Mortality rates remain negligible and major complications are rare. Given the technical success of this operation, the focus has more recently shifted away from objective surgical outcomes towards patient‐centred outcomes and satisfaction rates. Pleasingly, laparoscopic fundoplication has achieved consistently high rates of patient satisfaction [2].

Much work has been done refining techniques and comparing the different types of fundoplication, with the aim of improving both surgical and patient‐centred outcomes [3, 4, 5]. There are numerous randomised controlled trials and meta‐analyses comparing fundoplication types to identify the optimum operation [6, 7]. This has led to long‐term satisfaction outcomes upwards of 82% [8]. However, this also means that there remains 10%–20% of patients who are dissatisfied with the surgical management of their reflux disease [8].

Numerous variables have been identified as potential factors responsible for lower satisfaction after anti‐reflux surgery. This includes age and BMI [9]. Female sex is another variable often quoted as leading to lower satisfaction. In a paper looking at over 700 patients undergoing fundoplication, the authors concluded that females reported lower satisfaction after anti‐reflux surgery than men and had an almost double revision rate [10]. A similar conclusion was reached by Robertson et al. [3] where they found that females were twice as likely to report poor outcomes.

In 2017, our unit published a study looking at 225 patients undergoing anti‐reflux surgery [11]. The paper studied 13 symptoms in terms of who experiences them pre‐operatively, sex differences in symptom patterns and how those preoperative symptoms correlated with post‐operative dissatisfaction. Females reported higher post‐operative dissatisfaction than men in the study, with 15% of female patients not happy to recommend the surgery to others compared with only 10% of male patients. We concluded that the differing self‐reported symptom pattern was a large reason for differences in satisfaction rates. However, this symptom pattern was not clearly defined, and given almost all symptoms were experienced more by females than males, it was difficult to appreciate which symptoms were associated with the reported outcomes. Also, at that stage, we only looked at Nissen fundoplications (which represented the vast majority of patients in our practice at that time), so the impact of partial fundoplications was not yet known. With an additional 5 years of data as well as introducing partial fundoplications to the analysis, we believe we are better equipped to understand this vexing issue in upper gastrointestinal (GI) surgery.

It is our hypothesis that a female‐pattern of symptoms exists among patients presenting for surgery with GORD and our primary aim was to better define these symptoms using a more comprehensive method than in our previous study. We hypothesise that certain symptoms are more common in female patients and others are more common in male patients. A secondary aim is to see which symptoms or patterns of symptoms improved after surgery and to see if any symptoms improved more with a partial fundoplication compared to a Nissen fundoplication. We hypothesise that the patients with these particular symptoms would benefit more from a partial fundoplication than a Nissen fundoplication. Last, we hypothesise that partial fundoplication has become more common over the study period given the preceding hypothesis.

## 2 Methods

### 2.1 Study Design

This was a prospective observational cohort study.

### 2.2 Setting

De‐identified data were extracted from the audit database within the private practice of one of the authors (R.H.R.) following the format of previously published studies [8] The data reflected a consecutive series of patients undergoing laparoscopic hiatus hernia repair and fundoplication from 1998 until 2020.

### 2.3 Participants

Patients were offered fundoplication based on the combination of (typical) symptoms, response to antacid medication and findings on further investigations as recommended in the SAGES guidelines. Oesophagitis was considered as endoscopic proof of GORD, and increased oesophageal acidification (pH < 4.0 lasting more than 4% of the study period) and/or prolonged reflux episodes were classified as an abnormal 24‐h pH measurement study confirming GORD. Patients were denied anti‐reflux surgery if they presented with overtly atypical symptoms, significant oesophageal dysmotility observed on manometry concerning for the diagnosis of achalasia, or the identification of any other diagnosis that explained the patients' symptoms.

### 2.4 Surgical Interventions

Patients underwent either a total (Nissen) fundoplication or a partial (Toupet or Dor) fundoplication based on the surgeon's preference. This was influenced by patient preference after a discussion of the options and description of the relative merits of both types of fundoplication. The Nissen fundoplication was performed via a standard procedure including posterior crural repair and a 360° ‘floppy’ wrap over a 52‐Fr bougie after division of the fundal short gastric vessels. The partial fundoplication also involved a posterior crural repair and division of the fundal short gastric vessels, followed by a standardised posterior 270° wrap. A bougie was not used when performing a partial fundoplication. Due to the small number of patients undergoing an anterior 180° fundoplication (Dor)—performed only for patients with a diagnosed major oesophageal dysmotility—these patients were excluded from analysis. This was done to maintain homogeneity between the two groups and because any analysis on patients with anterior fundoplication as a unique group would be underpowered. One of the surgeons (R.H.R.) was present at all operations either as the primary or assisting surgeon.

### 2.5 Variables

The questionnaire assessed typical and atypical reflux symptoms as well as additional GI symptoms often seen in patients presenting for anti‐reflux surgery. (This last category was included as these symptoms are known to be more common among female patients and have well established links to functional GI complaints [12], that have been shown to influence patient outcomes in various fields of general surgery [13].) The typical reflux symptoms were: ‘Heartburn’ and ‘Regurgitation’. The atypical symptoms were: ‘Difficulty Swallowing’, ‘Chest Pain’, ‘Coughing’, ‘Asthma’ and the gastrointestinal symptoms were ‘Bloating’, ‘Excessive Wind’, ‘Belching’ and ‘Vomiting’. Also, from 2004 onwards, three additional GI symptoms were added to the questionnaire: ‘Abdominal Pain’, ‘Diarrhoea’ and ‘Constipation’. The only permitted responses for each symptom were ‘Frequently’, ‘Occasionally’, ‘Never’. The questionnaire was administered pre‐operatively and 18 months post‐operatively. These were the same questions with the addition of a satisfaction assessment on the post‐operative questionnaire. No further questionnaires were administered at later dates, given the evidence that patient outcomes following fundoplication plateau after 12–18 months [2, 14]. Patients who did not return their postoperative questionnaire were followed up by mail and by telephone and re‐invited to submit their responses.

### 2.6 Data Sources

De‐identified data were extracted from the audit database within the surgeons' private practice. The information obtained included basic demographics, operation details, pre‐operative questionnaire results as well as post‐operative questionnaire results.

### 2.7 Study Size

The power calculation was performed based on the primary outcome of predicting male patients and female patients from symptom patterns. WebPower was used on Rstudio [15] specifically with the wp.logistic function. Assuming a binary logistic model, 80% power, proportions of 0.4 vs. 0.6, two‐sided *α* = 0.05, and the predictor's distribution being Bernoulli, a sample size of 199 was required.

### 2.8 Statistics

Statistical packages for social sciences version 29 was used for analyses (SPSS ver 29, IBM, Armonk NY, USA [16]). For crosstabulation of discrete variables, Pearson Chi square tests (two‐sided) were used. *T*‐tests for independent means were used for comparing age. To predict which symptoms were more likely to occur in female patients, each individual symptom was tested in a binary logistic model controlling for age. A full multivariable model was then performed with all symptoms and age at once, using a backwards step procedure with criteria ‘PIN(0.05) POUT(0.20) ITERATE(20) CUT(0.5)’. For the date and percentage that were partial fundoplications, time was split into quintiles and Pearson Chi square test for independence was used.

## 3 Results

### 3.1 Patients

**FIGURE 1:**
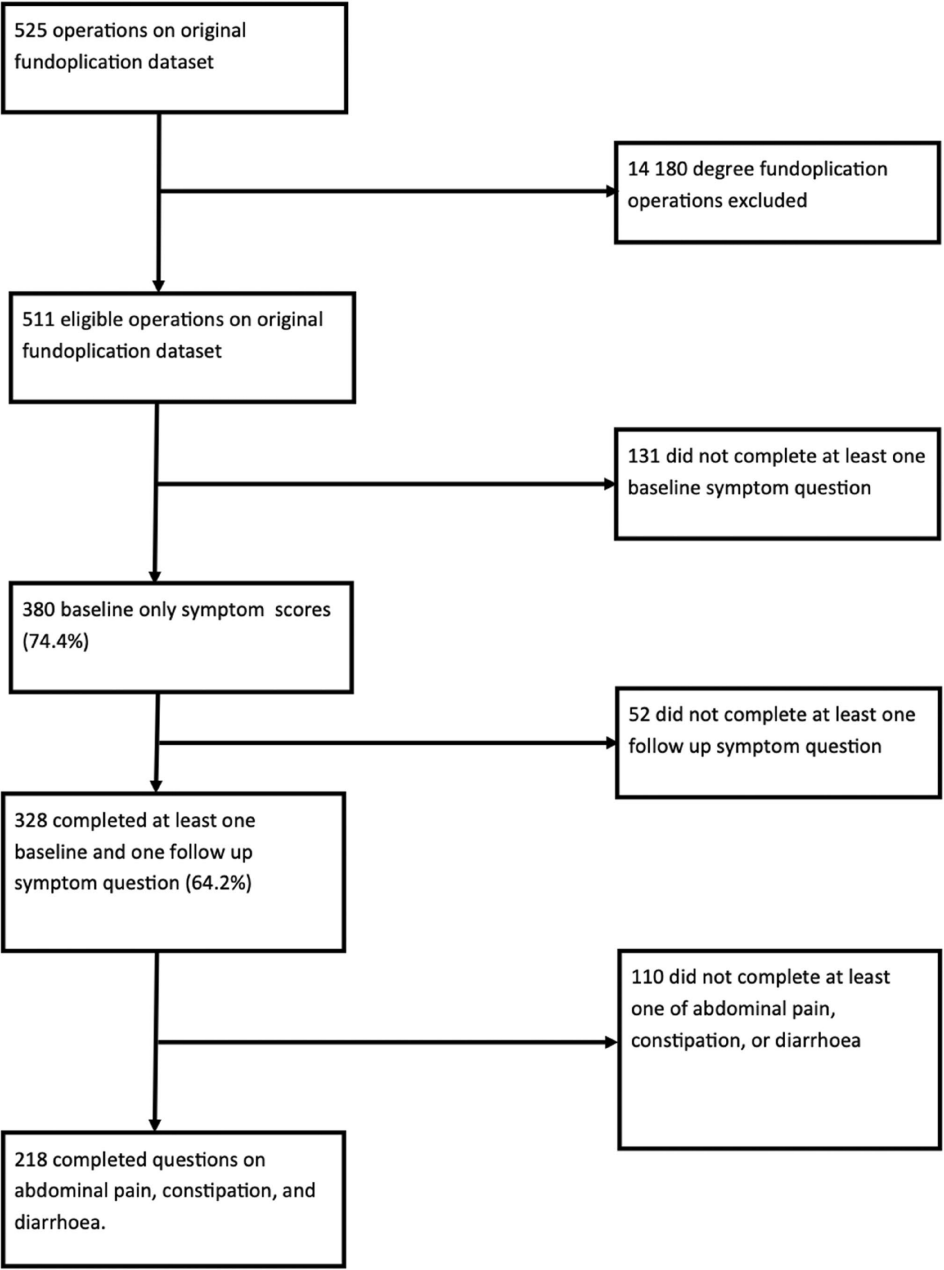
Participant flow.

Between 1998 and 2020, there were 525 operations (Figure 1). Fourteen patients underwent an anterior fundoplication and were therefore excluded. One hundred and thirty‐one failed to complete their baseline questionnaires and another 52 did not complete their follow‐up questionnaire. The 328 people remaining included 172 males and 156 females. Female patients' mean age was higher than male patients' (51.1 [SD = 12.4] F vs. 47.2 [SD = 13.3] M, *p* = 0.01). Fewer female patients underwent Nissen fundoplication compared with male patients (87 [55.8%] F vs. 135 [78.5%] M, *p* < 0.001). In total, 222 patients underwent a Nissen fundoplication (65.9%) and 115 patients underwent a posterior 270° fundoplication. People who received a Nissen fundoplication were younger than those who received a partial fundoplication (47.4 [12.6] vs. 52.5 [13.4], *p* < 0.001).

### 3.2 Pre‐Operative Symptoms Patterns

Table 1 describes which symptoms were experienced more by female patients vs. male patients. When controlling for age, individual pre‐operative symptoms which occurred significantly more in female patients were heartburn (*p* = 0.012), regurgitation (*p* < 0.001), bloating (*p* < 0.001), excess wind (*p* = 0.013), vomiting (*p* < 0.001), asthma (*p* = 0.036), abdominal pain (*p* = 0.008), constipation (*p* < 0.001) and diarrhoea (*p* < 0.001). In backwards step regression controlling for age wherein all symptoms were entered at once, bloating (*p* ≤ 0.001), asthma (*p* = 0.045), constipation (*p* = 0.010) and diarrhoea (*p* = 0.023) were significantly more associated with being female. The only pre‐operative symptom which was significantly associated with being male was chest pain (*p* = 0.023).

**TABLE 1:** Odds ratios predicting female sex.

	*n*	Frequency	Controlling for age only			Controlling for age plus all other symptoms (backwards step)		
			Odds ratio	95% Confidence interval	Omnibus *p*	Odds ratio	95% Confidence interval	Omnibus *p*
Heartburn	21	Never	1 (ref)	—	0.012*	NA	NA	NA
	92	Occasionally	1.27	0.46–3.49	—	NA	NA	NA
	215	Frequently	2.48	0.95–6.46	—	NA	NA	NA
Regurgitation	45	Never	1 (ref)	—	< 0.001*	NA	NA	NA
	153	Occasionally	1.59	0.78–3.25	—	NA	NA	NA
	128	Frequently	3.36	1.62–6.97*	—	NA	NA	NA
Bloating	67	Never	1 (ref)	—	< 0.001*	1 (ref)	—	< 0.001*
	133	Occasionally	1.66	0.88–3.12		2.51	1.01–6.27	
	127	Frequently	4.10	2.15–7.81*		8.21	3.03–22.24	
Dysphagia	168	Never	1 (ref)	—	0.069	NA	NA	NA
	127	Occasionally	1.60	0.999–2.56		NA	NA	NA
	31	Frequently	1.98	0.90–4.33		NA	NA	NA
Excess wind	57	Never	1 (ref)	—	0.013*	NA	NA	NA
	176	Occasionally	1.28	0.68–2.38		NA	NA	NA
	93	Frequently	2.48	1.24–4.95*		NA	NA	NA
Belching	45	Never	1 (ref)	—	0.995	NA	NA	NA
	147	Occasionally	1.02	0.52–2.01		NA	NA	NA
	135	Frequently	0.99	0.50–1.97		NA	NA	NA
Chest pain	127	Never	1 (ref)	—	555	1 (ref)	—	0.023*
	154	Occasionally	1.01	0.63–1.63		0.38	0.18–0.79	
	45	Frequently	1.44	0.72–2.86		0.35	0.12–0.996	
Vomiting	211	Never	1 (ref)	—	< 0.001*	NA	NA	NA
	99	Occasionally	2.40	1.46–3.94*		NA	NA	NA
	17	Frequently	3.84	1.28–11.52*		NA	NA	NA
Coughing	99	Never	1 (ref)	—	0.225	NA	NA	NA
	143	Occasionally	1.57	0.92–2.67		NA	NA	NA
	85	Frequently	1.49	0.82–2.73		NA	NA	NA
Asthma	235	Never	1 (ref)	—	0.036*	1 (ref)	—	0.045*
	71	Occasionally	1.54	0.89–2.64		2.08	0.92–4.67	
	20	Frequently	3.17	1.16–5.66*		4.96	0.99–24.87	
Abdominal pain	83	Never	1 (ref)	—	0.008*	NA	NA	NA
	99	Occasionally	2.22	1.21–4.06*		NA	NA	NA
	36	Frequently	3.08	1.33–7.16*		NA	NA	NA
Constipation	95	Never	1 (ref)	—	< 0.001*	1 (ref)	—	0.01*
	95	Occasionally	2.27	1.25–4.10*		1.59	0.79–3.21	
	29	Frequently	10.24	3.21–32.70*		6.77	1.94–23.60	
Diarrhoea	88	Never	1 (ref)	—	< 0.001*	1 (ref)	—	0.023*
	113	Occasionally	2.52	1.41–4.51*		2.33	1.15–4.72	
	19	Frequently	5.86	1.76–19.45*		4.36	1.09–17.39	

* *p* < 0.05 (two sided).

### 3.3 Rates of Improvement and Deterioration of Each Symptom Depending on Type of Fundoplication

The rates of net improvement for dysphagia were higher in patients undergoing a partial fundoplication (*p* < 0.01), while bloating trended towards having more people who just stayed the same (*p* = 0.22). Heartburn and regurgitation both demonstrated improvement in the majority of patients, but there was no statistical advantage towards either type of fundoplication (Table 2).

**TABLE 2:** Symptom changes by type of fundoplication.

Symptom	Frequency	Partial		Full		*p*
		*n*	%	*n*	%	
Heartburn	Improve	76	72.4%	161	74.9%	0.50
	Same	27	25.7%	46	21.4%	
	Worse	2	1.9%	8	3.7%	
Regurgitation	Improve	71	68.3%	155	73.8%	0.56
	Same	29	27.9%	47	22.4%	
	Worse	4	3.8%	8	3.8%	
Bloating	Improve	46	43.8%	98	46.0%	0.22
	Same	49	46.7%	82	38.5%	
	Worse	10	9.5%	33	15.5%	
Dysphagia	Improve	27	25.7%	50	23.4%	0.01*
	Same	61	58.1%	95	44.4%	
	Worse	17	16.2%	69	32.2%	
Excess wind	Improve	26	24.8%	46	21.5%	0.65
	Same	51	48.6%	101	47.2%	
	Worse	28	26.7%	67	31.3%	
Belching	Improve	49	47.1%	107	51.4%	0.74
	Same	42	40.4%	75	36.1%	
	Worse	13	12.5%	26	12.5%	
Chest pain	Improve	45	42.9%	79	38.0%	0.70
	Same	52	49.5%	113	54.3%	
	Worse	8	7.6%	16	7.7%	
Vomiting	Improve	33	31.4%	61	29.0%	0.67
	Same	65	61.9%	139	66.2%	
	Worse	7	6.7%	10	4.8%	
Coughing	Improve	55	51.9%	101	47.6%	0.33
	Same	41	38.7%	98	46.2%	
	Worse	10	9.4%	13	6.1%	
Asthma	Improve	15	14.7%	38	18.1%	0.46
	Same	81	79.4%	165	78.6%	
	Worse	6	5.9%	7	3.3%	
Abdo pain	Improve	31	31.3%	34	30.9%	0.75
	Same	53	53.5%	63	57.3%	
	Worse	15	15.2%	13	11.8%	
Constipation	Improve	36	35.6%	35	30.7%	0.74
	Same	57	56.4%	69	60.5%	
	Worse	8	7.9%	10	8.8%	
Diarrhoea	Improve	28	28.0%	35	30.4%	0.92
	Same	56	56.0%	63	54.8%	
	Worse	16	16.0%	17	14.8%	

* *p* < 0.05 (two sided).

### 3.4 Type of Fundoplication Over Time

Over the years there was a significant change in practice from initially performing predominantly Nissen fundoplications, towards more recently performing mostly partial fundoplications (*p* < 0.001). The 328 patients were divided into quintiles to demonstrate this point. The first quintile included 66 patients from 14 May 1998 to 2 October 2002, of which 92.4% underwent a Nissen fundoplication. While in the fifth quintile from 19 January 2017 to 7 October 2020, 92.3% of patients received a partial fundoplication. This trend is in keeping with a change in practice towards partial fundoplication that is evident in many centres around the world [17].

## 4 Discussion

Laparoscopic fundoplication is a safe and effective treatment for GORD with excellent overall patient satisfaction. The international literature reports effective treatment of heartburn in 80%–90% of patients for at least 10 years, with low complication rates and an acceptable side effect profile [18]. Furthermore, satisfaction rates of over 80% have consistently been reported in the literature [19]. Whilst these results are admirable, we believe we should continue to strive for even better results.

Amongst many variables, female sex has often been associated with decreased satisfaction rates, yet the reasons why this is true are poorly understood. Staehelin et al. [20] demonstrated that being female was associated with lower satisfaction after fundoplication and that this remained a significant factor on longer term follow‐up. Beck et al. [10] found that 82% of male patients were highly satisfied at 5 years post fundoplication, compared with only 77% for female patients; however, they too had no ‘clear explanation’ for these results.

In previous work [11] our unit alluded to a female‐symptom pattern in order to suggest that perhaps it was a particular constellation of symptoms rather than anything related to sex or gender per se that led to lower satisfaction rates. This would certainly be a nicer way of categorising patients, especially in an increasingly gender‐fluid environment. However, at that time we were not able to define this symptom pattern or explain which symptoms were most significant.

In this study we focused on the typical, atypical, and other GI symptoms that patients with GORD often present with, and were able to identify bloating, asthma, constipation and diarrhoea as being more common among female patients. Together with regurgitation, vomiting, excess wind and abdominal pain (which were significantly more likely to be female in the univariate analysis) we have identified a pattern of symptoms more commonly experienced by those of female sex. Whilst we acknowledge that GI symptoms are not directly related to oesophageal reflux, they often present concurrently in patients with GORD [21], and have been shown to lead to worse surgical outcomes in other conditions [13]. Therefore we believe that it is important to include these symptoms when defining a pre‐operative symptom pattern as they may affect patient satisfaction after anti‐reflux surgery.

Our paper then compared patients who underwent Nissen fundoplications to those who had a posterior 270° partial fundoplication. The debate surrounding the type of fundoplication has long waged in the literature. Historically, the focus has been on trying to identify a one‐size‐fits‐all approach, comparing and contrasting reflux control, durability and side effects depending on the type of wrap [22, 23]. An Australian study elegantly demonstrated that the higher the degree of wrap, the more durable the reflux control but also, the more likely the patient is to have side effects relating to the surgery [24]. Whilst many surgeons have changed practice to accept a potentially less durable partial fundoplication in preference to having to manage occasional side effects such as dysphagia from a tighter wrap [17], both techniques remain acceptable, effective and commonly utilised procedures in modern surgery [25].

There was some hope that partial fundoplication would be particularly effective in improving outcomes for female patients, or patients with the symptoms that we found to be more common among female patients. Abdominal pain, constipation and diarrhoea were expected to be such symptoms, given abdominal symptoms are often reported side effects following Nissen fundoplication [26]. Unfortunately, our study did not demonstrate any significant improvement for these patients with a partial fundoplication when compared with a Nissen fundoplication. In particular, we note that apart from dysphagia, performing a partial fundoplication did not lead to any greater improvement in any of the other symptoms compared with a Nissen fundoplication.

Several limitations exist in this study. As a prospective observational cohort study, the patients were not randomised and hence there may be some selection bias between the two types of fundoplication. However, as was demonstrated in this study, the change in practice, from doing almost exclusively Nissen fundoplications to almost exclusively partial fundoplications, does offer a mixing of patients and symptoms within the two groups. We also acknowledge the relatively short follow‐up time. We know that other studies have shown changes in symptoms beyond 10 years [19], several studies show that patient outcomes plateau beyond 12–18 months [2].

Ultimately, the issue with reduced satisfaction amongst female patients continues to trouble surgeons. Whilst we have shown a difference in symptom patterns between male and female patients, causality will be difficult to prove. Alternate hypotheses for female dissatisfaction have been proposed. It has been argued that female patients may be more inclined to report side effects or persistent symptoms whilst male patients have been socialised to neglect their problems and falsely report good outcomes [27]. This reporting bias is best described by Pilowsky's ‘illness behaviour’ in which they argue that a patient's experience of their illness (and treatment)—influenced by cultural and social factors—may contribute to satisfaction reporting to a greater extent than the actual illness itself [28]. Alternatively, satisfaction may be affected by confounding factors like anxiety—which are more common amongst female patients—and are known to lead to decreased satisfaction outcomes after anti‐reflux surgery [29].

In conclusion, there are certain symptoms that affect female patients with GORD more commonly than male patients. Whilst we cannot confirm the significance of this symptom pattern, it is reasonable to believe that these symptoms are contributing to the overall lower satisfaction among female patients. Unfortunately, partial fundoplication does not appear to offer any significant advantage in improving these specific symptoms, as had previously been hoped. We encourage surgeons to pursue a detailed patient history, including all GI symptoms, prior to offering anti‐reflux surgery and to consider the impact of these symptoms on patient outcomes. Furthermore, we advocate for further research into understanding satisfaction after anti‐reflux surgery, particularly among female patients.

## Author Contributions

**Joshua N. Hammerschlag:** conceptualization, methodology, project administration, validation, visualization, writing – original draft, writing – review and editing. **Ross H. Roberts:** conceptualization, data curation, investigation, methodology, project administration, resources, supervision, validation, visualization. **Andrew McCombie:** data curation, formal analysis, project administration, software, supervision, validation.

## Ethics Statement

Ethical approval was granted by the University of Otago Health Ethics Committee (reference HD20/041).

## Conflicts of Interest

The authors declare no conflicts of interest.
